# Characterization of the *Actinobacillus pleuropneumoniae* SXT-related integrative and conjugative element ICE*Apl2* and analysis of the encoded FloR protein: hydrophobic residues in transmembrane domains contribute dynamically to florfenicol and chloramphenicol efflux

**DOI:** 10.1093/jac/dkx342

**Published:** 2017-10-06

**Authors:** Yinghui Li, Yanwen Li, Roberto Fernandez Crespo, Leon G Leanse, Paul R Langford, Janine T Bossé

**Affiliations:** 1 Section of Paediatrics, Department of Medicine, Imperial College London, St Mary's Campus, London, UK; 2 Shenzhen Center for Disease Control and Prevention, Shenzhen, Guangdong, China

## Abstract

**Objectives:**

To characterize ICE*Apl2*, an SXT-related integrative and conjugative element (ICE) found in a clinical isolate of the porcine pathogen *Actinobacillus pleuropneumoniae*, and analyse the functional nature of the encoded FloR.

**Methods:**

ICE*Apl2* was identified in the genome of *A. pleuropneumoniae* MIDG3553. Functional analysis was done using conjugal transfer experiments. MIDG3553 was tested for susceptibility to the antimicrobials for which resistance genes are present in ICE*Apl2*. Lack of florfenicol/chloramphenicol resistance conferred by the encoded FloR protein was investigated by cloning and site-directed mutagenesis experiments in *Escherichia coli*.

**Results:**

ICE*Apl2* is 92660 bp and contains 89 genes. Comparative sequence analysis indicated that ICE*Apl2* is a member of the SXT/R391 ICE family. Conjugation experiments showed that, although ICE*Apl2* is capable of excision from the chromosome, it is not self-transmissible. ICE*Apl2* encodes the antimicrobial resistance genes *floR*, *strAB*, *sul2* and *dfrA1*, and MIDG3553 is resistant to streptomycin, sulfisoxazole and trimethoprim, but not florfenicol or chloramphenicol. Cloning and site-directed mutagenesis of the *floR* gene revealed the importance of the nature of the hydrophobic amino acid residues at positions 160 and 228 in FloR for determining resistance to florfenicol and chloramphenicol.

**Conclusions:**

Our results indicate that the nature of hydrophobic residues at positions 160 and 228 of FloR contribute dynamically to specific efflux of florfenicol and chloramphenicol, although some differences in resistance levels may depend on the bacterial host species. This is also, to our knowledge, the first description of an SXT/R391 ICE in *A. pleuropneumoniae* or any member of the Pasteurellaceae.

## Introduction

Integrative and conjugative elements (ICEs) are self-transmissible mobile genetic elements that play a major role in gene flow in bacterial populations and contribute greatly to antimicrobial resistance dissemination.^1^^,^^2^ Not only do ICEs tend to acquire insertion sequences encoding antimicrobial resistance genes that can then be conjugally transferred to other bacteria (including other species and genera), but the conjugal transfer machinery encoded by ICEs can also facilitate mobilization of small plasmids carrying resistance genes.^3–5^ Furthermore, ICEs and other conjugative plasmids can mediate retrotransfer, i.e. gene flow in two directions, with genes from the original recipient also transferring back to the original donor.^6–8^ Identification and characterization of ICEs is therefore important for understanding the mechanisms governing the flow of antimicrobial resistance genes, and other virulence factors, amongst bacterial populations.

With large numbers of bacterial genome sequences available for analysis, families of related ICEs are now being described and there is an online database (http://db-mml.sjtu.edu.cn/ICEberg) containing details of relevant features of known ICEs.^9^ To date, the SXT/R391 family of ICEs is the most extensively characterized, with 89 members currently listed in the ICEberg database. SXT was first discovered in MO10, a *Vibrio cholerae* O139 clinical isolate from India in the early 1990s, and carries genes encoding resistance to sulfamethoxazole, trimethoprim, chloramphenicol and streptomycin.^10^ R391 was first discovered in *Providencia rettgeri*, isolated in South Africa in 1967, and subsequently proved genetically and functionally to belong to the SXT family.^11^ The SXT/R391 ICEs share 52 highly conserved core genes that are involved in integration/excision, conjugative transfer and regulation.^12^ Element-specific phenotypes detected in SXT/R391 ICEs are normally conferred by the insertion of variable DNA sequences into five ‘hotspots’ (HS1–5) and four variable regions (VRI–IV).^12^ ICEs belonging to the SXT/R391 family have been identified in a number of γ-Proteobacteria, including *Proteus mirabilis*, *Providencia alcalifaciens*, *Shewanella putrefaciens* and *Photobacterium damselae* subsp. *piscicida*,^12–15^ but not previously in members of the Pasteurellaceae.

We recently identified ICE*Apl1*, a member of the ICE*Hin1056* family, carrying the *tet*(B) tetracycline resistance gene, in MIDG2331 and other serovar 8 clinical isolates of the swine respiratory pathogen *Actinobacillus pleuropneumoniae*.^16^ Here, we describe ICE*Apl2*, an SXT-related ICE carrying multiple resistance genes, in the chromosome of another *A. pleuropneumoniae* serovar 8 clinical isolate, MIDG3553. To our knowledge, this is the first SXT-related ICE identified in the Pasteurellaceae.

## Materials and methods

### Identification and sequence analysis of ICEApl2

MIDG3553 was isolated from the pneumonic lung of a pig in the UK in 2012 and identified as *A. pleuropneumoniae* serovar 8 by PCR as previously described.^17^ Genomic DNA was isolated from MIDG3553 using the FastDNA Spin Kit (MP Biomedicals) and shotgun WGS data were generated and assembled by the MicrobesNG Sequencing Facility (www.microbesng.uk). Sequence data were analysed using ResFinder,^18^ for identification of antimicrobial resistance genes, with thresholds of 98% identity and minimum length of 60%. Further sequence analysis was done using BLASTn and BLASTx. A comparative alignment was generated for sequences most similar to ICE*Apl2* using Mauve version 2015-02-25. Default parameters were used for all programs. The complete sequence of ICE*Apl2* has been deposited in GenBank (accession number MF187965).

### Conjugal transfer of ICEApl2

In order to investigate conjugal transfer of ICE*Apl2*, we used the same plasmid-free, nalidixic acid-resistant clinical *A. pleuropneumoniae* isolates as previously used to demonstrate conjugal transfer of ICE*Apl1*,^16^ namely MIDG3376 (serovar 6), MIDG2465 (serovar 7), MIDG3217 (serovar 8) and MIDG3347 (serovar 12). Furthermore, we also used the nicotinamide adenine dinucleotide (NAD)-independent ΔureC::nadV mutant of the serovar 8 strain MIDG2331, which contains ICE*Apl1*, as a recipient strain as previously described.^19^ Matings were performed as previously described,^20^ with selection on either brain heart infusion (BHI) agar supplemented with 0.01% NAD, 10** **mg/L trimethoprim and 20** **mg/L nalidixic acid or, in the case of the MIDG2331ΔureC::nadV recipient, on BHI agar (with no NAD) supplemented with 10** **mg/L trimethoprim. Selected transconjugants were tested by PCR for the presence of the *traG* gene using the primers listed in Table 1 and (where appropriate) for the presence/absence of *nadV* using primers described previously.^20^ Unless otherwise specified, reagents in the Qiagen Fast Cycling PCR Kit were used for all amplifications in this study. Selected transconjugants were tested for susceptibility to chloramphenicol and florfenicol, as described below.

**Table 1.** Primer sequences

Primer	Sequence 5′ to 3′	Target/purpose
traG_for	GAATAACCTTGGTTTGGCTTCGG	*traG*
traG_rev	CATACACGAGCATGAGCGAAATTG	
nadV_for	CTGTATGAGATTTAAGGAAAGAAATTATTATGGATAACC	*nadV*
nadV_rev	GCGTATTAAGTACAAATATCATAGCGTAGTGC	
floR_for	CGACGCCCGCTATGATCCAACTC	*floR* RT–PCR
floR_rev	CCCAAAAAGCCGGACTCGCGAAG	
IceFlo_L1_out	GCGAACGTCTTACCATCATTTCTC	circular forms and insertion sites of ICE*Apl2*
IceFlo_R1_out	GATTGGACTGAAATAGCGGAGG	
IceFlo_L2_out	GTGGTTTTAAGCGTTGAAAGGC	
IceFlo_R2_out	GGTAGCGAAGGAATTTGTGGTTTAG	
yhaH_5′_out	GCGTAAAGCATAGATAAACCACTCC	
prfC_5′_out	GCGAGATGATAGCAAAGGTCC	
FloR_start_for^a^	**GAGGG**TTGATTCGTC*ATGACCA*	cloning of *floR* genes
FloR_down_rev	GGATACCGACATTCACGAGGTTTC	
FloR_TAmut_for	CCGGTATGGGCACCTACTTCGTCTTCTTCTCG	site-directed mutagenesis
FloR_TAmut_rev	CGAGAAGAAGACGAAGTAGGTGCCCATACCGG	
FloR_CTmut_for	CCCTATCGCCGGAGTATTGATCGGCGAGTTC	
FloR_CTmut_rev	GAACTCGCCGATCAATACTCCGGCGATAGGG	

a In the sequence of primer FloR_start_for, the bold letters indicate the RBS and the italics indicate the start codon for the *floR* gene.

Chromosomal insertion sites were confirmed in MIDG3553, and in transconjugants, by PCR using the AccuPrime Taq DNA Polymerase High Fidelity Kit (Life Technologies), according to the manufacturer’s instructions. For the 5′ insertion, primers yhaH_5′_out and IceFlo_L1_out were designed to amplify a 561** **bp fragment from the *yhaH* gene (on the complement strand upstream from the ICE*Apl2* insertion) to a sequence within the 5′ end of ICE*Apl2*. For the 3′ junction, primers IceFlo_R1_out and prfC_5′_out were designed to amplify a 683** **bp fragment from within the 3′ end of ICEApl2 to a sequence within the downstream gene, *prfC*. Amplicons were sequenced using primers IceFlo_L2_out or IceFlo_R2_out, as appropriate.

### Confirmation of the circular extrachromosomal form of ICEApl2

DNA was extracted from MIDG3553 and selected transconjugants, and nested PCR was performed using the AccuPrime Taq DNA Polymerase High Fidelity Kit (Life Technologies), according to the manufacturer’s instructions. The outward-facing primers (IceFlo_L1_out and IceFlo_R1_out), used for detection of the insertion sites above, were paired to amplify a 983 bp fragment of the closed circular form of the ICE. To facilitate sequencing for identification of the cross-over point (*attP*), this product was used as template in a second PCR to amplify a 360 bp fragment using primers IceFlo_L2_out and IceFlo_R2_out, and the resulting amplicon was sequenced using primers IceFlo_L2_out and IceFlo_R2_out.

### Antimicrobial susceptibility testing

Antimicrobial susceptibility testing was performed by broth microdilution according to the CLSI VET01-A4 guidance.^21^ The antimicrobial agents tested were trimethoprim, sulfisoxazole, streptomycin, chloramphenicol and florfenicol, with MIC determinations performed at least twice on independent occasions.

### Comparison of floR expression in MIDG3553 and MIDG3446

RT–PCR was used to investigate transcription of *floR* in the *A. pleuropneumoniae* isolates MIDG3553 (carrying ICE*Apl2* in the chromosome) and MIDG3446 (a previously described isolate carrying the florfenicol resistance plasmid pM3446F).^22^ Expression levels were also investigated in the presence/absence of 2** **mg/L chloramphenicol in the culture medium. In brief, for each isolate, RNA was extracted from 1 mL of broth culture (OD_600_ = 1) using the RNeasy Mini Kit (Qiagen) as per the manufacturer’s instructions. Residual DNA was removed using the TURBO DNA-free Kit (Life Technologies). RNA (600 ng) was used as the template for cDNA synthesis using Invitrogen III First-Strand Synthesis SuperMix, as per the manufacturer’s instructions. The cDNA products of each sample (2 μL) were amplified using floR_for/floR_rev primers (Table 1) and the resulting 510 bp PCR products were visualized with ethidium bromide after separation by agarose gel electrophoresis.

### Cloning and mutagenesis of floR genes

The *floR* genes from MIDG3553 and MIDG3446 were amplified from genomic DNA using primers FloR_start_for/FloR_down_rev (Table 1) and the AccuPrime Taq DNA Polymerase High Fidelity Kit (Life Technologies), according to the manufacturer’s instructions. The 1300 bp PCR products, which included 15 identical bases upstream of the *floR* genes [containing the ribosomal binding site (RBS), but not the endogenous promoter], were purified using the QIAquick PCR Purification Kit (Qiagen), cloned into pGEM-T Easy Vector (Promega) and transformed into *Escherichia coli* Stellar™ competent cells (Clontech), following the manufacturers’ protocols. Three to five transformants were selected from BHI/ampicillin/IPTG/X-Gal or BHI plates containing 8 mg/L chloramphenicol. Plasmids were extracted from transformants using the QIAprep Spin Miniprep Kit (Qiagen), analysed by restriction enzyme digestion (SacII and RasI) to confirm correct size, and sequenced using primers FloR_start_for/FloR_down_rev (Table 1) to confirm fidelity of the cloned insert. Plasmids confirmed to have the correct inserts were designated p3446-*floR* and p3553-*floR*.

Site-directed mutagenesis was performed on p3553-*floR* using the QuikChange Lightning Site-Directed Mutagenesis Kit (NEB), with primers (see Table 1 for sequences) FloR_CTmut_for/FloR_CTmut_rev to change C to T at base 479, or FloR_TAmut_for/FloR_TAmut_rev to change T to A at base 683. The resulting plasmids, p3553-CT and p3553-TA, were transformed into Stellar™ competent cells (Clontech). Plasmids were extracted and the *floR* genes sequenced using primers FloR_start_for/FloR_down_rev. Subsequently, p3553-TA was further mutated to change C to T at base 683, as above, to generate p3553-CT/TA.

*E. coli* clones containing each of the above plasmids were tested for susceptibility to chloramphenicol and florfenicol, as described above, and compared with the control *E. coli* containing no plasmid.

## Results and discussion

### Identification and characterization ICEApl2

**Figure 1.**
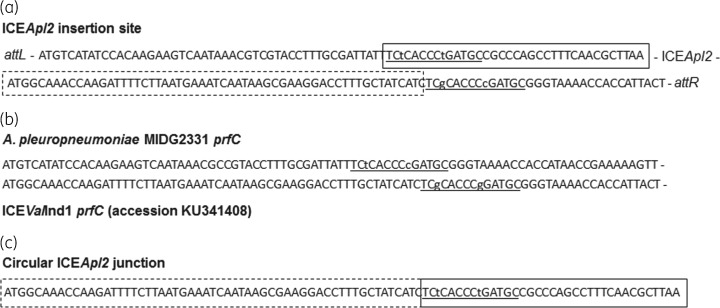
(a) The imperfect DRs (underlined, with lower-case letters denoting the bases that differ) flanking ICE*Apl2* share sequence identity with 14 bases found in *prfC* genes (b) from *A. pleuropneumoniae* (e.g. accession number CUU51349) and *Vibrio* species (e.g. accession number KU341408). (c) The closed circular form of ICE*Apl2* shows that the junction (*attP* sequence, underlined) formed by cross-over between the repeats retains a 14 bp sequence differing only at one base from the *A. pleuropneumoniae prfC* gene. Sequences in the closed and dashed boxes indicate the 5′ and 3′ ends, respectively, of the ICE*Apl2* sequence.

As for SXT/R391 ICEs found in other species,^12^^,^^23^ ICE*Apl2* is inserted into the *prfC* gene in *A. pleuropneumoniae* strain MIDG3553. Analysis of the sequences bordering the left and right attachment sites of ICE*Apl2* in the chromosome of MIDG3553 showed an imperfect 14 bp repeat (with differences at two bases; Figure 1a), similar to sequences found in *prfC* genes from *A. pleuropneumoniae* and SXT-containing species such as *Vibrio alginolyticus* strain ANC4-1 (Figure 1b). Sequencing of the closed circular form of ICE*Apl2* shows the junction formed by cross-over between these repeats retains the 14 bp sequence (*attP*; Figure 1c) differing only at one base from the *A. pleuropneumoniae prfC* gene (Figure 1b).

**Figure 2.**
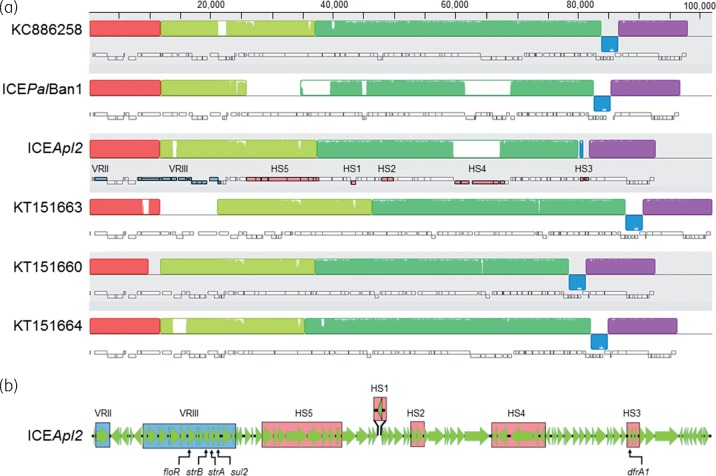
Analysis of the ICE*Apl2* sequence. (a) Mauve alignments of ICE*Apl2* and closely related members of the SXT/R391 ICE family. For ICEs from *V. cholerae* isolates, accession numbers are shown (accession numbers KC886258, KT151663, KT151660, KT151664). Syntenic regions of conservation are shown by the larger rectangular blocks. The orientation and relative size of genes in each ICE are indicated by the small rectangular blocks (lower blocks are on the complementary strand), with the accessory genes present in VRII–III and HS1–5 of ICE*Apl2* highlighted as in the larger view of this ICE shown in (b). This figure appears in colour in the online version of *JAC* and in black and white in the print version of *JAC*.

Initial BLASTn analysis of the complete ICE*Apl2* sequence and subsequent Mauve alignments (Figure 2a) revealed extensive regions of similarity with numerous SXT-R391 ICEs,^12^^,^^24^ including ICE*Pal*Ban1 from *P. alcalifaciens* (accession number GQ463139) and ICEs from various *V. cholerae* strains (e.g. accession numbers KT886258, KT151660, KT151663, KT151664). In addition to the conserved backbone of core genes found in all SXT-R391 ICEs, most of the accessory genes found in ICE*Apl2* (Figure 2a and b) have been reported in other members of this family.^12^^,^^25^ In all SXT/R391 ICEs characterized to date, accessory genes are found in five hotspots (HS1–5), with up to four further variable regions (VRI–IV) depending on the ICE, all in conserved locations along the backbone.^12^^,^^25^^,^^26^

ICE*Apl2* contains accessory genes that map to all five hotspots (HS1–5), as well as VRII and VRIII (Figure 2b). As in ICE*Pal*Ban1 and ICEs from certain *V. cholerae* strains,^12^^,^^24^ there is a single gene insertion in VRII encoding a predicted DNA mismatch repair protein, and an IS*CR2*-related insertion in VRIII that includes the antimicrobial resistance genes *floR*, *strAB* and *sul2*. The nine-gene insertion in HS5 (seven predicted restriction modification genes and two hypothetical genes) is the same as that found in some *V. cholerae* ICEs (e.g. accession number KT151663), as well as in the *P. mirabilis* R997 ICE (accession number KY43363). HS1 also contains the same single-gene insertion (encoding a predicted replicative DNA helicase) as the *P. mirabilis* R997 ICE.^25^ The toxin/antitoxin genes found in HS2 in ICE*Apl2* are the same as found in this position in numerous SXT-R391 ICEs.^12^

There are five genes in HS4 of ICE*Apl2*, the first four of which do not match sequences in other known SXT-R391 ICEs. BLASTx searches indicated the first gene encodes a P-loop NTPase common to Enterobacteriales (accession number WP_060434990); the second encodes a predicted transposase that is most closely related to a sequence from *Rheinheimera* sp. KL1 (accession number WP_053424104); and the third and fourth genes are most similar to two contiguous genes found in *Shewanella* sp. Sh95 (accession numbers WP_055648497 and WP_055648496), encoding a predicted DEAD-box helicase and a hypothetical protein, respectively. Only the fifth gene in this region, encoding a predicted endonuclease, is common to numerous SXT ICEs found in *V. cholerae* strains (including accession numbers KT151664, KY382507 and KC886258), as well as in ICE*Psp*Spa1 found in *Pseudoalteromonas* sp. 4mar (accession number HG794404).

HS3 in ICE*Pal*Ban1 contains the same seven genes, including the trimethoprim resistance gene *dfrA1*, as found in some *V. cholerae* ICEs.^12^ Of these, only the *dfrA1* gene is found in this site in ICE*Apl2* and it is on the opposite strand compared with the other ICEs. A further two hypothetical genes are found downstream of *dfrA1* in HS3 of ICE*Apl2*, the first of which shows greatest identity with a gene from *Rheinheimera* sp. KL1 (accession number WP_053425140) and the second of which shows no significant homology with any known genes in GenBank.

### Conjugal transfer of ICEApl2

Four *A. pleuropneumoniae* clinical isolates representing serovars 6, 7, 8 and 12 were used to test the conjugal transfer ability of ICE*Apl2*; however, no transconjugants were obtained. The isolates tested (MIDG2465, MIDG3217, MIDG3347 and MIDG3376) had been used successfully as recipients for conjugal transfer of ICE*Apl1*, with conjugation frequencies of 10^−4^–10^−5^, both in this and the previous study.^16^ A large number of genes are involved in conjugal transfer,^27^^,^^28^ and no attempt was made to determine the reason for lack of conjugation of ICE*Apl2* into these strains.

**Figure 3.**
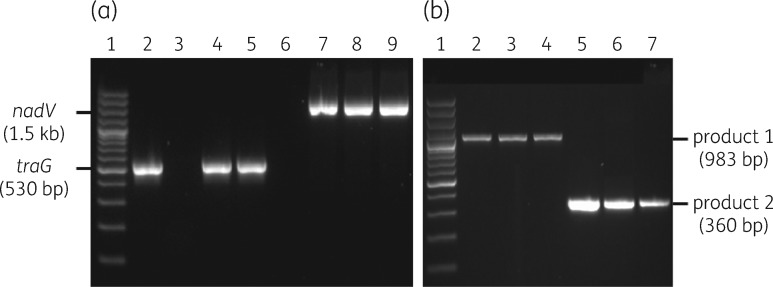
PCR confirmation of ICE*Apl2* in MIDG2331ΔureC::nadV and detection of extrachromosomal ICE*Apl2* in MIDG3553 and MIDG2331ΔureC::nadV. (a) PCR products from amplification of *traG* (530 bp) and *nadV* (1.5 kb) sequences from donor MIDG3553 (lanes 2 and 6), recipient MIDG2331ΔureC::nadV prior to conjugation (lanes 3 and 7) and selected transconjugants (lanes 4, 5, 8 and 9). Lane 1, 100 bp Plus DNA ladder. (b) Detection of a circular intermediate form by nested PCR in MIDG3553 (lanes 2 and 5) and selected transconjugants (lanes 3, 4, 6 and 7). Nested-PCR products generated by primers IceFlo_L1_out/IceFlo_R1_out and IceFlo_L2_out/IceFlo_R2_out are labelled as product 1 (983 bp) and product 2 (360 bp), respectively. Lane 1, 100 bp Plus DNA ladder (Invitrogen).

Transconjugants were obtained when MIDG2331ΔureC::nadV, harbouring ICE*Apl1*, was used as the conjugal recipient for ICE*Apl2*, although the conjugation frequency was only 10^−7^. Selected transconjugants were confirmed as MIDG2331ΔureC::nadV containing ICE*Apl2*, as PCR showed detection of both the *traG* (found in ICE*Apl2*) and *nadV* genes (Figure 3a). The presence of a circular intermediate form of ICE*Apl2* was confirmed both in MIDG3553 and in selected transconjugants by nested PCR (Figure 3b). These results suggest that, although ICE*Apl2* is capable of excision from the chromosome, it is not self-transmissible. Successful conjugation into MIDG2331ΔureC::nadV suggests that ICE*Apl1* present in this strain mediated retrotransfer of ICE*Apl2*. This phenomenon, first described in 1987 by Mergeay *et al.*,^6^ was shown to require prior transfer of a conjugal plasmid from donor to the recipient, followed by a second separate DNA transfer event back to the donor strain.^7^ Retrotransfer has been shown to meditate mobilization of both plasmids and chromosomal segments from recipient to donor at low frequencies.^6^^,^^7^ We previously used MIDG2331ΔureC::nadV as the recipient strain to demonstrate mobilization of small trimethoprim resistance plasmids, pM3389T and pM3224T, from their respective donor strains, MIDG3389 and MIDG3224.^19^ Although MIDG3389 was later shown to also harbour ICE*Apl1*,^16^ there is no evidence of conjugal transfer genes in the genome of MIDG3224 (accession number ERS134355)^29^ and it is likely that the ICE*Apl1* in MIDG2331ΔureC::nadV mediated retrotransfer of the pM3224T plasmid, although this was not appreciated at the time.^19^

### Antimicrobial susceptibility of MIDG3553

Antimicrobial susceptibility testing of MIDG3553 confirmed resistance to trimethoprim, sulfisoxazole and streptomycin (Table 2). However, MICs of florfenicol and chloramphenicol (0.5 and 1 mg/L, respectively) indicated susceptibility to these antimicrobial agents that are normally exported by the FloR efflux protein. When plated on increasing concentrations of chloramphenicol, it was possible to select colonies of MIDG3553 resistant to 2 mg/L of this antimicrobial agent (MIC = 4 mg/L), but no increased resistance was found for florfenicol. In contrast, *A. pleuropneumoniae* isolates MIDG3446 and MV518, with identical *floR* genes on plasmids pM3446F and p518 (respectively), were both shown to have MICs of florfenicol and chloramphenicol ≥8 mg/L.^22^^,^^30^ Furthermore, the MIDG2331 transconjugants containing ICE*Apl2* also had MICs of 4 mg/L of chloramphenicol and 0.5 mg/L of florfenicol, suggesting that these low MICs are related to the *floR* sequence encoded by ICE*Apl2* and are not specific to the *A. pleuropneumoniae* isolate harbouring the ICE.

**Table 2.** Antimicrobial resistance genes located in ICE*Apl2* and levels of resistance to relevant antimicrobial agents for *A. pleuropneumoniae* MIDG3553

Antimicrobial resistance gene in ICE*Apl2*	Antimicrobial agent	MIC (mg/L)
*dfrA1*	trimethoprim	≥1024
*sul2*	sulfisoxazole	≥1024
*strA*, *strB*	streptomycin	256
*floR*	florfenicol	0.5^a^
	chloramphenicol	1^b^

a Not considered resistant; MICs of florfenicol ≥8 mg/L are considered resistant for *A. pleuropneumoniae*.^21^

b There is no published breakpoint for resistance of *A. pleuropneumoniae* to chloramphenicol; however, plasmids pM3446F and p518, carrying identical *floR* genes, both conferred MICs of florfenicol and chloramphenicol of 8 mg/L for their respective *A. pleuropneumoniae* isolates, MIDG3446 and MV518.^22^^,^^30^

**Figure 4.**
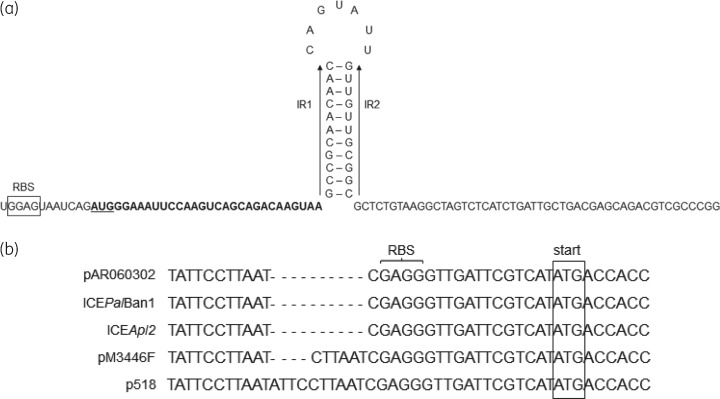
Comparison of sequences upstream of the *floR* gene present in selected plasmids and ICEs. (a) Sequence corresponding to a putative translational attenuator found immediately upstream of all sequences shown in (b). The putative attenuator includes a stem–loop containing perfect IR sequences (labelled IR1 and IR2), preceded by a short ORF encoding a peptide of nine amino acids (bold type), with the corresponding RBS shown boxed. (b) Differences detected in the sequences from *A. pleuropneumoniae* plasmids pM3446F and p518, compared with those in the *E. coli* plasmid pAR060302, as well as ICE*Pal*Ban1 and ICE*Apl2*, were restricted to tandem duplications of 6 and 10 bp (respectively), upstream of the RBS and the *floR* start codon (box). The sequences shown in (b) are continuous with that shown in (a).

Lang *et al.*^31^ reported that exposure of *E. coli* carrying the multiresistance plasmid pAR060302 to florfenicol resulted in an 8-fold increase in expression of *floR*, indicating inducible expression of this gene; however, they did not explore the nature of the regulation involved. Other florfenicol and/or chloramphenicol resistance genes (*catA*, *cmlA* and *fexA*) have been shown to be inducible by chloramphenicol and have similar translational attenuator sequences that overlap the RBS of the associated resistance genes.^32–34^ Analysis of the region upstream of the *floR* genes in ICE*Apl2* and pAR060302 revealed they are identical and contain a sequence that shares many of the features of the attenuators previously reported for the other chloramphenicol-inducible genes.^32–34^ The putative *floR*-associated attenuator sequence (Figure 4a) includes a short ORF encoding a peptide of nine amino acids, followed by perfect 11 bp IR sequences (labelled IR1 and IR2) that could form a stem–loop structure. However, this structure does not overlap the RBS of the *floR* gene, which is located further downstream. A BLASTn search confirmed that this putative attenuator sequence is well conserved (100% nucleotide identity) upstream of *floR* genes in many plasmids and ICEs, including those in the *A. pleuropneumoniae* plasmids pM3446F and p518. However, differences can be seen just upstream of the *floR* RBS in pM3446F and p518, comprising tandem duplications of either 6 or 10 bases, respectively (Figure 4b). It is not apparent how, if at all, these small insertions would contribute to differences in *floR* expression.

**Figure 5.**
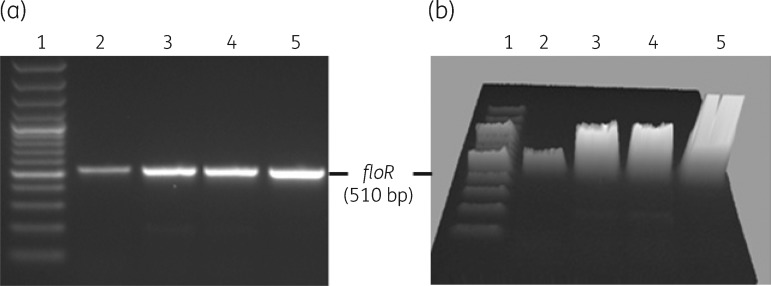
Expression of *floR* in MIDG3553 (lanes 2 and 3) and MIDG3446 (lanes 4 and 5) as determined by RT–PCR amplification of a 510 bp sequence. Lanes 2 and 4 show results for samples grown in broth without chloramphenicol and lanes 3 and 5 show results for samples grown in broth with 2 mg/L chloramphenicol. Lane 1, 100 bp Plus DNA ladder (Invitrogen). (a) Normal 2D gel image. (b) Gel image shown in 3D.

RT–PCR was used to compare expression levels of *floR* in MIDG3553 and MIDG3446, with and without chloramphenicol induction. The results shown in Figure 5 indicate that the *floR* gene was expressed in MIDG3553, though at a lower level than in MIDG3446. This is possibly due to differences in copy number of the *floR* gene found on the plasmid pM3446 (in MIDG3446) compared with that in the chromosome as part of ICE*Apl2* in MIDG3553. Growth in the presence of 2 mg/L chloramphenicol increased expression of the *floR* gene in both isolates (Figure 5). However, it should be noted that, even with increased expression of *floR* in MIDG3553 grown in the presence of chloramphenicol, there was no increase in resistance to florfenicol, indicating a possible difference in the function of the FloR protein encoded by ICE*Apl2* compared with that by pM3446F.

Comparative analysis of the *floR* genes from ICE*Apl2* versus pM3446 revealed two base differences at positions 479 and 683, associated with conservative hydrophobic amino acid substitutions at positions 160 and 228 in the resulting proteins. The FloR protein, a member of the major facilitator superfamily (MFS) of drug:H+ antiporter proteins, contains 12 transmembrane segments.^35^ The FloR encoded by pM3446 has a valine (V) residue at position 160 and a tyrosine (Y) residue at position 228, whereas the FloR encoded by ICE*Apl2* has an alanine (A) residue at position 160 and phenylalanine (F) at position 228. Although all four of these amino acids are hydrophobic, alanine and valine are aliphatic, whereas phenylalanine and tyrosine are aromatic.^36^ A prediction of membrane topology of the FloR efflux protein^35^ indicates that these two positions are located in the fifth and sixth putative transmembrane segments, TMS5 and TMS6, though residue 160 may be accessible to the periplasm. Site-directed mutagenesis of membrane-embedded charged residues previously identified D23, in TMS1, and R109, in TMS4, which contribute to recognition of phenicol derivatives, and E283, in TMS9, which may be necessary for correct folding of the protein.^35^ These authors only investigated charged residues that could affect proton translocation or substrate recognition, whereas the residues at positions 160 and 228 in both the pM3446F- and the ICE*Apl2*-encoded FloR proteins are hydrophobic amino acids. A recent study of the *E. coli* MdtM multidrug exporter, another MFS protein, indicated that hydrophobic residues Y57 and F253 form an ‘aromatic clamp’ around the neutral chloramphenicol substrate, whereas the Y26 and Y123 residues form a similar clamp around the cationic tetraphenylphosphonium (TPP^+^) substrate.^37^ Mutagenic exchange of the tyrosine and phenylalanine residues in a Y26F/F253Y double mutant was found to abrogate TPP^+^ efflux, but had no effect on chloramphenicol efflux.^37^

In the current study, we used cloning and site-directed mutagenesis to investigate the relative resistance to florfenicol and chloramphenicol in *E. coli* conferred by FloR proteins differing in the hydrophobic residues found at positions 160 and 228. The *floR* genes (with identical upstream sequences including the endogenous RBSs, but not the promoters) from *A. pleuropneumoniae* MIDG3553 and MIDG3446 were cloned into a high-copy plasmid (pGEMT Easy) under control of the plasmid’s promoter in order to detect differences due to the respective encoded proteins, rather than differences in expression level. The cloned sequences differed only at the two bases in the *floR* gene encoding the amino acids at positions 160 and 228. The p3446-*floR* plasmid (FloR with V160 and Y228) conferred MICs of florfenicol and chloramphenicol of 512 and 256 mg/L, respectively, whereas p3553-*floR* (FloR with A160 and F228) conferred the same level of resistance to chloramphenicol, but 3 logs lower resistance to florfenicol, in *E. coli* (Table 3). Although the higher MICs of florfenicol and chloramphenicol detected for the *E. coli* clones compared with the *A. pleuropneumoniae* isolates carrying the same respective *floR* genes are likely due, at least in part, to the copy number of the plasmid used for cloning, the patterns of resistances detected differed between the bacterial hosts. It is not clear why the two FloR proteins confer equal resistance to chloramphenicol in *E. coli*, but not in *A. pleuropneumoniae*, whereas differences in florfenicol resistance are seen in both species.

**Table 3.** Cloned *floR* genes from *A. pleuropneumoniae* isolates MIDG3446 and MIDG3553, which differ at two bases, confer different levels of resistance to florfenicol and chloramphenicol in *E. coli*

*floR* plasmid	Base (479)^a^	AA (160)^b^	Base (683)	AA (228)	MIC of florfenicol (mg/L)^c^	MIC of chloramphenicol (mg/L)
p3446-floR	T	V	A	Y	512	256
p3553-floR	C	A	T	F	64	256
p3553-CT	T	V	T	F	64	32
p3553-TA	C	A	A	Y	128	256
p3553-CT/TA	T	V	A	Y	512	256
No plasmid^d^					16	8

a Base at the corresponding position (shown in parentheses) in the *floR* sequence.

b Amino acid at the corresponding position (shown in parentheses) in the FloR sequence.

c There is no published breakpoint for resistance of *E. coli* to florfenicol; however, strains carrying the *floR* gene have been reported to have MICs ranging from 16 to ≥ 256 mg/L.^32^ A CLSI breakpoint of ≥ 32 mg/L of chloramphenicol is indicated for *E. coli*.^21^

d *E. coli* Stellar with no plasmid.

Site-directed mutagenesis was used to determine the relative contributions of the amino acids at positions 160 and 228 to resistance to florfenicol and chloramphenicol in *E. coli* (Table 3). The combination of V160 and F228 encoded by plasmid p3553-CT (with A160V mutation of the p3553-*floR* encoded protein) resulted in the same level of florfenicol resistance as conferred by p3553-*floR*, but decreased resistance to chloramphenicol by 3 logs to an MIC of 32 mg/L. In contrast, the combination of A160 and Y228, encoded by p3553-TA, resulted in the same chloramphenicol resistance, but 1 log higher resistance to florfenicol, compared with that conferred by p3553-*floR*. Mutation of both residues in p3553-CT/TA, resulting in expression of FloR with V160 and Y228, conferred the same levels of resistance as p3446-*floR*, with the same sequence. From these results, it appears that the presence of phenylalanine (which carries a hydroxyl side group not found on tyrosine) at position 228 of FloR impedes efflux of florfenicol, but not chloramphenicol, whereas the presence of valine rather than alanine at position 160 affects efflux of chloramphenicol, but only when in combination with phenylalanine at position 228. It has been previously noted that valine is a poor, whereas alanine is a good, helix-forming residue.^38^ This may, in combination with the hydroxyl side chain found on phenylalanine, impede the efflux of chloramphenicol by FloR with V160 and F228.

It is clear from these results that the nature of the hydrophobic residues at positions 160 and 228 in the transmembrane segments of FloR contributes dynamically to substrate recognition and transport in *E. coli*. The nature of the amino acids at these positions may also, at least in part, contribute to the lack of resistance to florfenicol and chloramphenicol observed in *A. pleuropneumoniae* MIDG3553. Further research will help define how these and possibly other hydrophobic residues in the various transmembrane domains contribute to substrate recognition and efflux by this MFS family protein in *A. pleuropneumoniae* and other bacterial species.

### Summary

We have identified ICE*Apl2*, a member of the SXT/R391 ICE family, in a serovar 8 clinical isolate of the swine respiratory pathogen *A. pleuropneumoniae*. To our knowledge, this is the first time a member of this ICE family has been reported in any member of the Pasteurellaceae. We demonstrated that ICE*Apl2* is capable of excision from the chromosome, but is impaired for self-mobilization; retrotransformation was confirmed to a recipient harbouring ICE*Apl1*. We demonstrated that the FloR protein encoded by ICE*Apl2* does not confer resistance to florfenicol or chloramphenicol in *A. pleuropneumoniae*, but does in *E. coli*. Furthermore, we identified, for the first time to our knowledge, that the nature of the hydrophobic residues at positions 160 and 228 of FloR contributes dynamically to specific efflux of florfenicol and chloramphenicol in *E. coli*. Our results not only inform on the structure/function of the FloR protein, but also demonstrate that, at least for *A. pleuropneumoniae*, presence of a *floR* gene does not necessarily indicate resistance to florfenicol/chloramphenicol. Molecular surveillance for florfenicol resistance in *A. pleuropneumoniae* therefore requires sequencing rather than PCR detection of the *floR* gene.
